# Understanding gift-giving in game live streaming on Douyu: An evaluation of PSR/social presence

**DOI:** 10.3389/fpsyg.2022.953484

**Published:** 2022-08-11

**Authors:** Wenchi Zhang

**Affiliations:** Department of Sociological Studies, The University of Sheffield, Sheffield, United Kingdom

**Keywords:** game live streaming, streamers, gift-giving, para-social relationship, Douyu, social presence

## Abstract

In China, live streaming has grown rapidly in recent years, with gift-giving, a unique business model in live streaming, driving the development of many industries. This article explores the association between gift-giving behavior in game live streaming and viewers' live streaming experience. Specifically, this study aims to explore the correlation between *Para-social Relationships, Social Presence*, and gift-giving in the context of China. Based on the survey and interview of the viewer on Douyu, a Chinese live streaming platform, this study found that there is only a weak to a medium correlation between para-social relationships and viewers' gift-giving behavior. The correlation between social presence and gift-giving was even weaker. Although the conclusion of this study may be affected by the sample size limitation, it can still provide a reference for the current research on gift-giving on Chinese live streaming platforms.

## Introduction

Over the last decades, the interest of viewers in user-generated content and live streaming have rapidly increased due to the development of the Internet and media technologies (Hilvert-Bruce et al., 2018). Distinct from traditional streaming media such as radio and TV, live streaming media allows media content to be used by viewers simultaneously as transmission (Sjöblom and Hamari, 2017). Furthermore, live streaming provides more opportunities for viewers to interact with content creators, also known as streamers (Gandolfi, 2016). Specifically, live streaming involves streamers presenting their behavior to viewers in real-time, either *via* video or audio. Viewers can then comments on the streamers' behavior or interact directly with the streamers through the real-time comments function on the platform (Lim et al., 2020). Based on these two characteristics, which differ significantly from traditional streaming media, scholars also define *live streaming* as synchronous or interactive media (Wohn et al., 2018).

Live streaming was not widely used initially, and its origins can be traced back to video game players. In order to share the gameplay, the players used live streaming to communicate with each other (Li and Guo, 2021). This phenomenon inspired many young people affected by the financial crisis, who started to engage in the live streaming industry and make a living from games (Guarriello, 2019; Johnson and Woodcock, 2019a). The participation of these young people has driven the development of live streaming. In addition, the popularity of professional Esports has helped democratize the game live streaming, which ultimately has a positive impact on the overall live streaming industry.

Although contemporary live streaming has developed to include many non-game categories, such as outdoor adventure, beauty, and gourmet cooking (Wohn et al., 2018), game live streaming still accounts for a large proportion of its use. Scholars also considered it one of game culture's most significant elements (Li et al., 2018). Take Twitch, currently the most popular live streaming platform in Europe and the United States, as an example (Sjöblom et al., 2019). According to an industry report, nine of Twitch's top 10 most-watched categories in 2021 were video games, with a combined 9.163 billion hours of watching (StreamElements, 2021).

There is a unique business model in live streaming: virtual gift giving, also known as donating, involves viewers giving streamers gifts. Giving virtual gifts on Chinese live-streaming platforms is especially popular with viewers (Li and Guo, 2021). Case in point, there were 7.3 million viewers who sent virtual gifts on Douyu, a Chinese live-streaming platform, in the fourth quarter of 2021. Since virtual gifts usually require viewers to spend real money to buy them, gift-giving provides financial support for streamers and ensures the operation and development of live streaming platforms (Johnson and Woodcock, 2019b; Li and Peng, 2021). Many live streaming platforms have launched unique virtual currencies as conversion systems between real money and virtual gifts. For instance, Twitch launched the “Bits” virtual currency in 2016. Viewers who want to give gifts need to purchase Bits with real money, then use Bits to buy the platform's virtual gift “Cheers”, and finally give the “cheer” to the Streamer (Bennett, 2022). It is worth noting that virtual gifting is a voluntary behavior, and viewers can also watch live streaming without sending a virtual gift (Yu et al., 2018; Zhang et al., 2019). Although gift-giving gives streamers much income, some scholars believe that streamers may be distracted by excessive gifts from the viewer, leading to a decline in the quality of live streaming content. In addition, the streamer's desire for gifts may increase because of the audience's gift-giving behavior (Wang et al., 2019).

Existing studies have identified motivations behind the viewer's gift-giving behavior. For instance, viewers motivated by social integration contributed more virtual gifts for live streaming content creators (Sjöblom and Hamari, 2017; Hilvert-Bruce et al., 2018). Specifically, the motivation of social integration involves the viewer's desire for a more satisfying community experience and more adequate social interactions in live streaming. In essence, viewers seek to establish and maintain different social relationships with content creators and other viewers by giving virtual gifts. These relationships include “Para-social relationships”(PSR), which refers to the imaginary friend-like relationship between viewers and media content creators; and “Social Presence” (SP) which refers to viewers' perception of other viewers in the live streaming.

Current researchers have mainly focused on the Twitch platform. Research on virtual gift-giving on Chinese live streaming platforms is insufficient. Furthermore, there is still a lack of research on gift-giving behavior in game live streaming. Therefore, this research aims to explore the correlation between PSR, SP, and gift-giving in Chinese game live streaming. Through the thematic analysis of the survey and interview data, this research found that neither PSR nor SP was significantly associated with gift-giving behavior. In comparison many viewers gave gifts out of appreciation and admiration of content creators' game skills. The remaining chapters of this paper will first review the theories and findings of PSR and SP in the existing literature, and then the research methods will be elaborated. Finally, the results of this study will be presented and discussed.

## Literature review

There are two main income sources for content creators in streaming media. The first is the traditional business revenue model, which relies on advertising and sponsorship. The second is the social revenue model, which relies on donations and the gift-giving behavior of viewers (Sjöblom et al., 2019). Existing studies which focus on viewers' motivation to use and participate in streaming media have identified a correlation between financial support behavior and viewers' motivation for social integration, such as an interest in deeper community engagement and a more satisfactory community experience (Sjöblom and Hamari, 2017).

The desire for community experiences is also a theme of the research of Hilvert-Bruce et al. (2018). They applied the Uses and Gratification theory to study the relationship between social motivation and live streaming participation. They found that the viewers' live streaming subscriptions and donations positively correlated with their need for social interaction and a sense of community. They also found that social interaction and a sense of community were stronger within smaller streaming channels. These two findings indicated that viewers who actively participating in smaller channels were more likely to donate and give gifts to the streamers they watched. However, this latter finding was also a limitation of the research because the social motivation model adopted in this study could not effectively explain users' live streaming participation in different channels. Specifically, this model only significantly interpreted viewers' live streaming participation of those who preferred smaller channels. Consequently, it could not effectively explain the participation of the viewers who preferred larger channels, particularly as participation of viewers in larger channels represents the majority of live streaming usage (Kaytoue et al., 2012). Therefore, the results of this study may lack representativeness.

In order to have a macroscopic and comprehensive understanding of the majority of live streaming usage, many researchers now use PSI and PSR to study the relationship between viewers and streamers. These terms were first proposed by Horton and Richard (1956) to describe phenomena in traditional media, but which also exist in social media (Jin and Park, 2009; Cohen and Tyler, 2016). Furthermore, PSR and PSI are often conflated in academia and beyond (Leith, 2021).

PSI is the way viewers interact with traditional media figures or celebrities such as hosts, talk show actors, soap operas, and comedy actors (Rubin and Perse, 1987; Auter, 1992). It gives viewers the illusion that the interaction is face-to-face (Houlberg, 1984). When PSI continues to occur, a PSR will be developed between the viewers and the traditional media figures (Rihl and Wegener, 2019). PSR refers to the imaginary friend-like relationship between viewers and traditional media figures (Rubin et al., 1985; Klimmt et al., 2006). It increases viewers' attachment and loyalty (Xiang et al., 2016). PSI, by contrast, represents the “reception behavior” of users in media or an instance in which media figures have the power to interact. PSI is typically one-sided (Li et al., 2021). The power of communication and interaction between users and media figures is unequal (Horton and Richard, 1956)—an inequity made evident by the observation that media figures' responses to user interactions are often absent (Dibble et al., 2016).

In the social media environment, traditional media figures and celebrities appear on social media due to media viewers' expectations (Chin, 2018). Meanwhile, social media offers traditional media figures and celebrities more control over what they present or disclose (Colliander and Dahlén, 2011). Therefore, traditional media figures and celebrities can create an enormous but carefully filtered amount of seemingly authentic self-disclosure in an online environment (Marwick and Boyd, 2011). These self-disclosures enhance viewers' engagement with media figures and celebrities (Frederick et al., 2014). It also enhances viewers' perceived intimacy. As a result, PSR has gained new development on social media (Chung and Cho, 2017).

The development of PSR on social media can also be reflected in the possibility of more two-way interaction between viewers and traditional media figures or celebrities (Click et al., 2013). Viewers are motivated by these possibilities and then engage in more PSI (Sanderson, 2009), leading to stronger PSR between viewers and media figures or celebrities (Tsiotsou, 2015). Although traditional media figures can choose to remain in control of one-sided interactions (Kehrberg, 2015), viewers are still free to follow the social media accounts of those media figures. For example, viewers can like, retweet, and comment on their social media posts without having a response (Yan and Yang, 2021). These affordances have been identified by scholars as “parasociability”.

The possibility of developing PSR on social media is not unique to traditional media figures or celebrities. Micro-celebrities also get the opportunity to develop PSR with their followers (Abidin, 2015). The term “micro-celebrity” was initially used by Senft (2008) to describe individuals who use blogs, videos, and other social network platforms to gain public attention and fame on the Internet. Such individuals can be public figures looking to acquire a stronger public image or even ordinary social media users (Khamis et al., 2017). Some scholars believe that the term “micro-celebrity” is not just a term applied to a person, but also describes the process of how (and why) ordinary social media users and public figures become famous through technology on digital platforms (Driessens, 2013; Usher, 2020). There are many alternatives to the term “Micro-celebrity” such as “social media influencer” (Kay et al., 2020), “digital Influencer” (Cotter, 2019), “Content Creator” (Arriagada and Ibáñez, 2020), and, most simply, “Influencer” (King and de la Hera, 2020).

Influencers cultivate their relationships with followers through self-disclosure on social media, in ways similar to traditional media figures and celebrities (Bishop, 2021). Specifically, they make textual or visual disclosure of their life or lifestyle on social media (Abidin, 2015), that can seem authentic and raw (Abidin, 2016). Influencers also cultivate intimacy between themselves and followers through methods such as communicating with followers in a friendly tone, or making fun of their own embarrassing moments, a tactic that highlights that they are as everyday ordinary as their followers (McQuarrie et al., 2013). Cultivating intimacy lays the foundation for an emotional connection between influencers and users (Marwick, 2013). Consequently, the construction of authenticity and intimacy positively enhances the PSR between influencers and users.

In the context of live streaming, the affordances provided by the platform enable streamers to respond to viewers' interactions while presenting media content (Leith, 2021), which makes the interaction between streamers and users “authentic” rather than “imagined” (Wulf et al., 2020). However, such interactions are not common, where the initiator of interaction is the viewers. Therefore, PSR still effectively represents the relationship between users and media figures in live streaming (Wulf et al., 2021).

Previous research has confirmed that PSR in streaming media positively correlates with the viewer's financial support, including donation and gift-giving behavior for streamers (Wohn et al., 2018). Yu et al. (2018) show an example of this. They found in their study that users who contributed many donations and virtual gifts to the streamer did not pay much attention to other streamers. They exclusively focused their attention on one or a few streamers.

Research has also discovered that the viewers who made many donations and display gift-giving behavior use more chat functions than other viewers on the streamer's channel. These viewers also made more attempts to establish a relationship with the streamer. The same result appeared in Zhu et al. (2017), who found a positive correlation between the frequency of viewers participating in the channel chats and gift-giving behaviors. In other words, the more viewers use chat functions, the more likely they support the streamer they are chatting to financially.

The two previous research results are also the embodiment of PSR to improve the attachment and loyalty of viewers (Xiang et al., 2016). The research of Hilvert-Bruce et al. (2018) has thus been supplemented. However, the method used in smaller channels is still questionable. In small channels, the interaction by viewers received by streamers is not exponential, so streamers can effectively interact with the viewer (Leith, 2021). This interaction pattern is distinct from the imagined, one-sided interaction described in PSR and distinct from media personalities controlling the power of interaction in the past (Rubin et al., 1985). Viewers obtained more interaction power in small live channels. The effectiveness of interaction makes the interaction between the viewer and the streamer no longer depend on imagination, so the relationship between viewers and streamers in small channels is closer to real-world relationships than para-social relationships (Rihl and Wegener, 2019).

Another factor that influences viewers' donation and gift-giving behavior is the social presence perceived by users in streaming media (Lin, 2021). This feeling highlights the individuals and their interpersonal interaction (Short et al., 1976). In live streaming, the unique user interface shows real-time viewing numbers, gifts given, endorsements of streamers, and other elements which embody SP. Not only do viewers perceive others in these elements, but they can also be perceived by other viewers (Lin, 2021). Additionally, the interaction between streamers and viewers, such as streamers will express their gratitude to the gift-giver publicly on the channel, which also strengthens the viewer's experience of SP (Yu et al., 2018).

It is worth noting that previous studies have demonstrated a positive association between SP and PSR. For instance, Kim and Song (Kim and Song, 2016) investigated the effect of celebrity self-disclosure on followers on Twitter. Their results showed that self-disclosure by stars enhanced their fans' experience of social presence, and this enhanced experience was positively correlated with the PSR between fans and stars. Most previous studies showing a positive correlation between SP and PSR have focused on social media such as Twitter. The results from these studies could not effectively interpret the situation in live-streaming, as they focused on the social media posts, rather the live-streaming.

This study aims to explore the motivations of gift-giving by viewers in the context of game live streaming in China *via* two research questions:

- Is there a positive or negative correlation between PSI/PSR in game streaming and gift-giving?
- Is there a positive or negative correlation between social presence in game streaming and gift-giving?

## Methods

This research examined the viewer's and gift-giving behavior to streamers in game live streaming by utilizing mixed methods. Specifically, this research adopted the Sequential Explanatory approach. The researcher began with a quantitative study: questionnaires. Then the researcher used the qualitative research method of the interview to verify and expand the data in the quantitative research. The advantage of this method is that the data analysis has more depth (Tashakkori and Teddlie, 2021).

The detail of the methods will be discussed in the following sections.

### Douyu

The researcher adopted Douyu, a streaming media platform, as the case of this research. Douyu platform originated from Acfun, an animation game portal. In 2013, Acfun attempted to create a live streaming website called Acfun Namah oso, considered an early prototype of Japanese live streaming. Then in 2014, Acfun relaunched the service, and the new website was named Douyu (Zhang and Hjorth, 2019).

There are three reasons for choosing Douyu. First of all, the researcher is a long-term user of Douyu and is familiar with the platform, such as the classification of streaming media channels in Douyu and the memes in some large channels. Secondly, Douyu fits in well with the purpose of the research. There are a large number of virtual gifts in Douyu. The main gifts can be divided into two categories. The first category of gifts is free gifts, such as “like”, “fan glow stick”, and so on. As long as viewers complete specific tasks on the platform, they can get a certain number of these free gifts, such as watching a certain number of streamers and watching a certain length of the live streaming. Another gift category requires the viewer to spend real currency to purchase, such as “plane” “rocket”. Also, gifts that cost more real money to buy will have a more significant visual effect and stay longer on the live page, visible to all viewers on the channel.

The third reason for choosing Douyu is that it has a “fan club” and a hierarchical aristocratic identity system for its viewers. The “fan club” function is similar to Twitch subscriptions, in which viewers receive a fan badge in front of their user name after giving a specific gift to the streamer: a “card”. Badges can be upgraded by increasing intimacy with the host. Douyu's aristocratic identity system functions like a membership system with ranks. Viewers need to charge real money to reach these levels, and as the level increases, viewers will earn more equity. These benefits include, but are not limited to: a unique banner above the live screen when the user enters a channel; users will have a dedicated channel assistant; users have the right to push a channel directly to Douyu's “Recommendation” interface. The presence of these elements, such as more colorful gift effects and stronger identity display, makes PSR and SP more accessible to the audience.

### Questionnaires

In this study, the researcher adopted a mixed approach. The first method implemented was the questionnaire in quantitative research. Because the users of live streaming are mainly young people (Chen and Lin, 2018), and Douyu was selected as the research object in this study. Therefore, purposive sampling was utilized to recruit respondents. This sampling method makes the recruited participants more consistent with the research questions and requirements through the strategic sampling of participants (Clark et al., 2019).

In light of Gaiser and Schreiner (2009) suggestion that playrooms or interactive gaming technologies can be considered as potential recruitment places for young people with certain technical knowledge and communication skills, participants were recruited in the Douyu community in Baidu Tieba. Baidu Tieba is an online communication platform similar to Reddit that allows users to form and join different online communities based on different interests (Zhao et al., 2020).

The performance parameters of this research were divided into two parts. Firstly, in order to ensure the accuracy of the data, all the recruited participants must meet the condition that they have followed at least one Douyu game streamer. Secondly, for the questionnaire and interview, the parameter of gift-giving behavior was set as the total value of the total gifts. Since other parameters cannot effectively fit this research. For example, some participants might be used to giving cheap or free gifts. In this case, the participants may have given so many gifts that the total number of gifts could not be easily calculated, but the participants could easily recall the total value of the gifts. The recruitment of participants lasted for 10 days, a total of 298 completed surveys were received; 267 were validated. Most respondents were 18–25; 164 identified as women and 103 as men.

### Interview

In order to verify the data obtained in the questionnaire and the depth of the research, the researcher conducted follow-up interviews with some questionnaire participants. The interview is a method in qualitative research, which is suitable for generating theories because it tries to generate data about how people perceive and understand specific aspects of the real world, and these data are abundant (Clark et al., 2019). Specifically, semi-structured interviews were utilized in this research. Although in semi-structured interviews, researchers will set a series of open questions in advance (DiCicco-Bloom and Crabtree, 2006), these preset questions will also change with the dialogue between researchers and interviewees. Some new questions may emerge from the dialogue (Bryman, 2016).

Ten interviewees were randomly selected from the questionnaire participants, and they all expressed a willingness to be interviewed. The duration of each interview was between 10 and 20 minutes. Furthermore, due to geographical and time constraints, all interviews in this research were conducted on Wechat, a Chinese social media. This type of interview potentially protects the interviewee since the interviewee's personal or social characteristics are not transparent to the researcher (Bowker and Tuffin, 2004).

### Data analysis

Since the questions in the questionnaire are single-choice and multiple-choice, the researcher used the Goodness of fit test to analyze the data of the questionnaire. This method can effectively analyze whether there is a significant difference in the proportion of each option selected. The data of the interview were coded and analyzed in Nvivo, using thematic analysis. This analysis method assists researchers in identifying and analyzing recurring themes and patterns in the research (Braun and Clarke, 2006; Vaismoradi et al., 2013). It presents rich details in the data and effectively “explains” the research themes (Nowell et al., 2017). Moreover, the thematic analysis approach adopted in this research is inductive. This coding method has no preset topic framework, and data drive the summarized themes and patterns. The same approach can be found in King and de la Hera (2020). They also utilized semi-structured interviews and thematic analysis to study viewers' perceptions of game streamers.

The six steps of thematic analysis proposed by Braun and Clarke (2006) were adopted in this study. They were as follows:

- Get familiar with the initial data;
- Create the initial code;
- Search for themes;
- Review the established themes;
- Name and define the theme;
- Report final results.

## Findings and discussions

In order to confirm the correlation between PSR and gift-giving on Douyu, the existence of PSR needs to be verified first. Most participants reported their illusion of intimacy with game streamers based on the questionnaire results. The measurement of PSR in this questionnaire was designed based on Rubin and Perse's (1987) scale, although there are other ways to measure PSR in academia. For example, the EPSI Scale proposed by Hartmann and Goldhoorn (2011). This method is more suitable for measuring the PSR of viewers and media figures in traditional media streams. Furthermore, the PSR scale proposed by Tukachinsky (2010). This scale contains 24 predictors involving Para-social friendship and Para-social love, which are beyond the scope of this research. Consequently, the scale of Rubin and Perse (1987) was adopted. Specific details will be discussed in the following part.

### The existence of PSR on Douyuz

**Figure 1:**
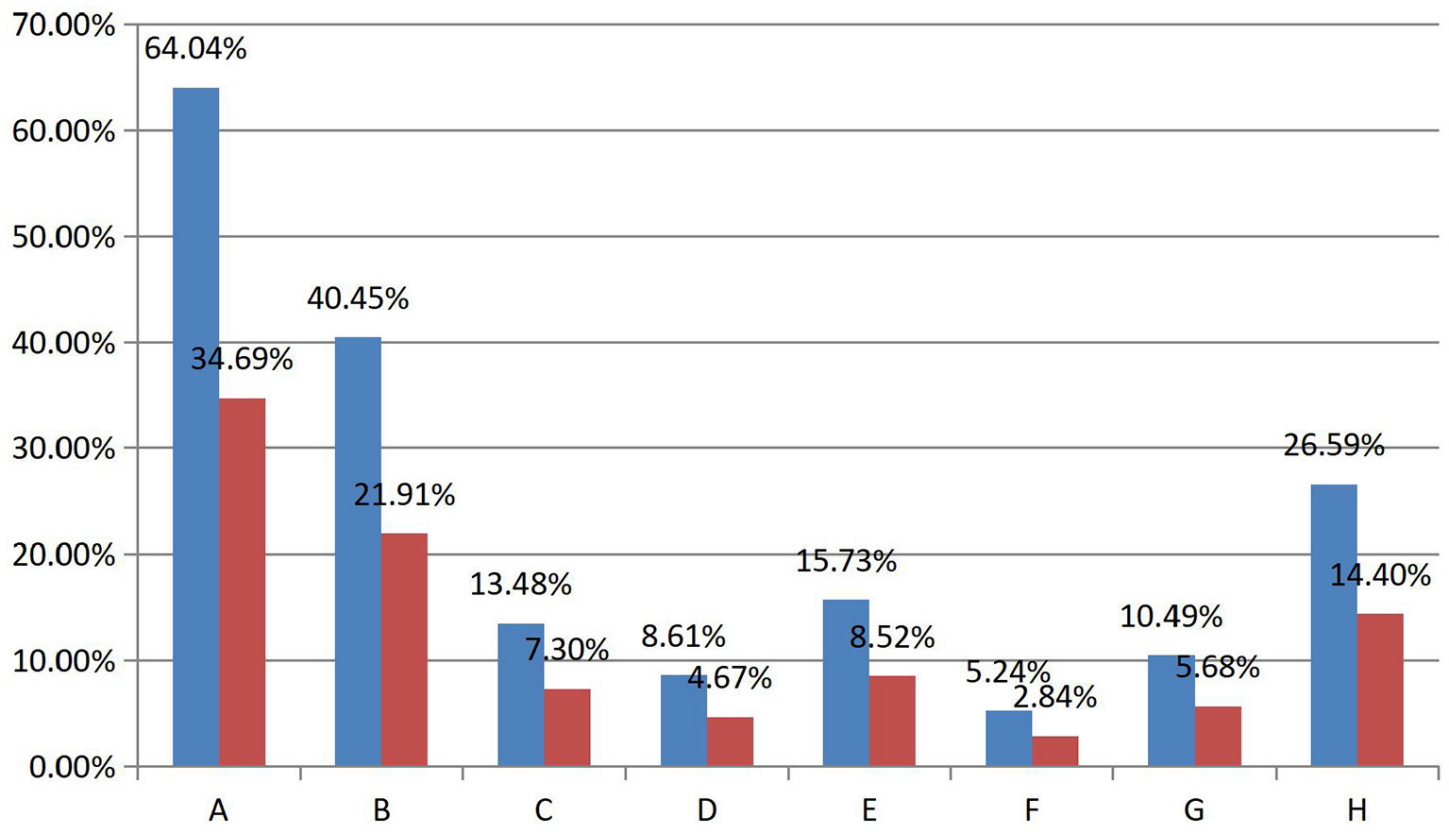
Summary table of and prevalence rates. (A) Someone who is talented. (B) Someone who is authentic. (C) Someone who is sophisticated. (D) Someone who is distant. (E) Someone I admire. (F) Someone who understand me. (G) Someone who is the person I want to meet in person. (H) Someone who is my friend (blue bar is prevalence rates, red bar is response rates. See Table 1 in the Appendix for specific values).

In the questionnaire, there were eight prediction options that measured the existence of PSR, five of which were adapted from the prediction model established by previous scholars (see Figure 1). They have weak to medium response rates respectively in this study. First, the highest response rate among the five prediction options was the viewer's perception of game streamers' authenticity, with a response rate of one in five (*N* = 493). The remaining four options had a combined response rate of 30 percent. They are: viewers think “streamer is someone they admire”; “Someone who understands them”; “A person who wants to meet in person”; And “someone like a friend”. The overall response rate of these five options was over 50 percent, proving that PSR exists between the viewers and game streamers on Douyu.

Two of the three remaining prediction options deny PSR's presence on Douyu: Viewers perceive the game streamer as “Someone sophisticated” and “Someone distant”. However, the combined response rate of these two options was just over 10 percent, which is not representative. Furthermore, one of the remaining indicates viewers' recognition of game streamers' technical prowess in live streaming, with a one in three response rate. In conclusion, although the response rate of the remaining prediction options accounted for nearly 50 percentages, the total response rate of the two options, which predicted the non-existence of PSR, was insignificant, and the other option could not strongly predict the existence of PSR. Therefore, the finding that PSR exists between viewers and game streamers on Douyu still holds.

During the interview, the author asked the interviewees about their choices in the questionnaire. For the PSR prediction option with the highest response rate, “Viewers perceive streamers as someone who is authentic”, respondents' replies focused on game streamers' performance during live streaming. The author named these recurring patterns and themes “Interactions that break the boundaries of fantasy.” by the author. For example, one of those interviewed wrote:

“*The Streamers I watch often interact with the viewers… He can see what viewers have said in the real-time comment system, and he will select some of them to respond. And sometimes his responses to the audience go on for a while.” (participant 6)*

This statement confirms Wulf's findings that the game streamer's interaction with the viewers during the live streaming is authentic, bringing the viewer into a “dialogue”. This “dialogue” has two dimensions. First, it refers to the fast, two-way connection (interaction) between viewers and streamers. Viewers are immersed in this connection and are encouraged to stay longer in this live streaming. In other words, this dimension of dialogue represents the increasing possibility of a two-way connection between the viewer and the game streamer, and these possibilities motivate the viewer to engage in more PSI (Sanderson, 2009).

The second dimension of “dialogue” refers to the game streamer's online community. When the viewer stays in the first “dialogue” for a long time, they may develop an emotional attachment to the game streamer and develop a deceptive intimacy with the game streamer, thus becoming a member of the game streamer's online community (Xiang et al., 2016). This dimension is a manifestation of the PSR. In conclusion, these two dialogue dimensions demonstrated that long-term PSI ultimately fosters PSR between viewers and game streamers (Click et al., 2013; Tsiotsou, 2015).

However, not all game streamers can consistently interact with their viewers during live streaming. The author questioned participants who responded in the survey that they perceived game streamers as “someone who is distant.” The survey results showed that respondents who chose this prediction option did not have a strong sense of the game streamer's interaction. As one noted in the interview:

“*The streamer I watch regularly has too many followers; his channel is very popular every day… Almost every second, dozens of new messages appear in the real-time comment system… He may not be able to handle such a large amount of information while playing.” (Participant 9)*

Unlike the patient and persistent interactions of game streamers reported by Participant 6, participant 9 did not have a strong sense of the game streamer's interaction. A key reason for such differences lies in the different reputations of the game streamers that the two participants often watch, which can also be understood as related to the size of the audiences on different live-streaming channels. Specifically, participant 6's most-watched game streamer has only moderate popularity on the Douyu, while Participant 9's most-watched game streamer has over 19 million subscribers on Douyu. The channels of game streamers that are extremely popular have numerous viewers participating at all times. As a result, even if the game streamers want to interact with viewers, the sheer volume of chats makes it impossible for the most popular streamers to engage effectively with any or all of their viewers. The researcher named this recurring pattern and theme as “Always control the power of interaction”—such situations confirm that PSI is typically one-sided and controlled by the streamer.

Although the absence of two-way connections in large channels may affect PSI, PSR is noted to occur still. The reason is that the game streamers utilize different methods and tactics to maintain a sense of intimacy with their viewers during live streaming, ultimately leading to PSR. For example, as another interviewee noted:

“*It is funny to watch his live streaming… I like his style of discourse, which has a sense of humor and at the same time stands on its own. There are many memes about his words in our community of followers… And, obviously, you can see a lot of awkward moments, and for the most part, he makes fun of his own awkward moments. That is what I like about him.” (Participant 1)*

The response of Participant 1 outlines a way game streamers cultivate intimacy with their viewers—by communicating with their viewers in a friendly, accessible manner, for example, by making fun of their embarrassing moments (McQuarrie et al., 2013). The cultivation of intimacy lays the foundation for an emotional connection between the game streamer and the viewers, ultimately leading to the viewers' PSR toward the game streamer. In conclusion, PSR was confirmed to exist on Douyu, across large and small sites.

#### The correlation between PSR and gift-giving behavior on Douyu

**Figure 2:**
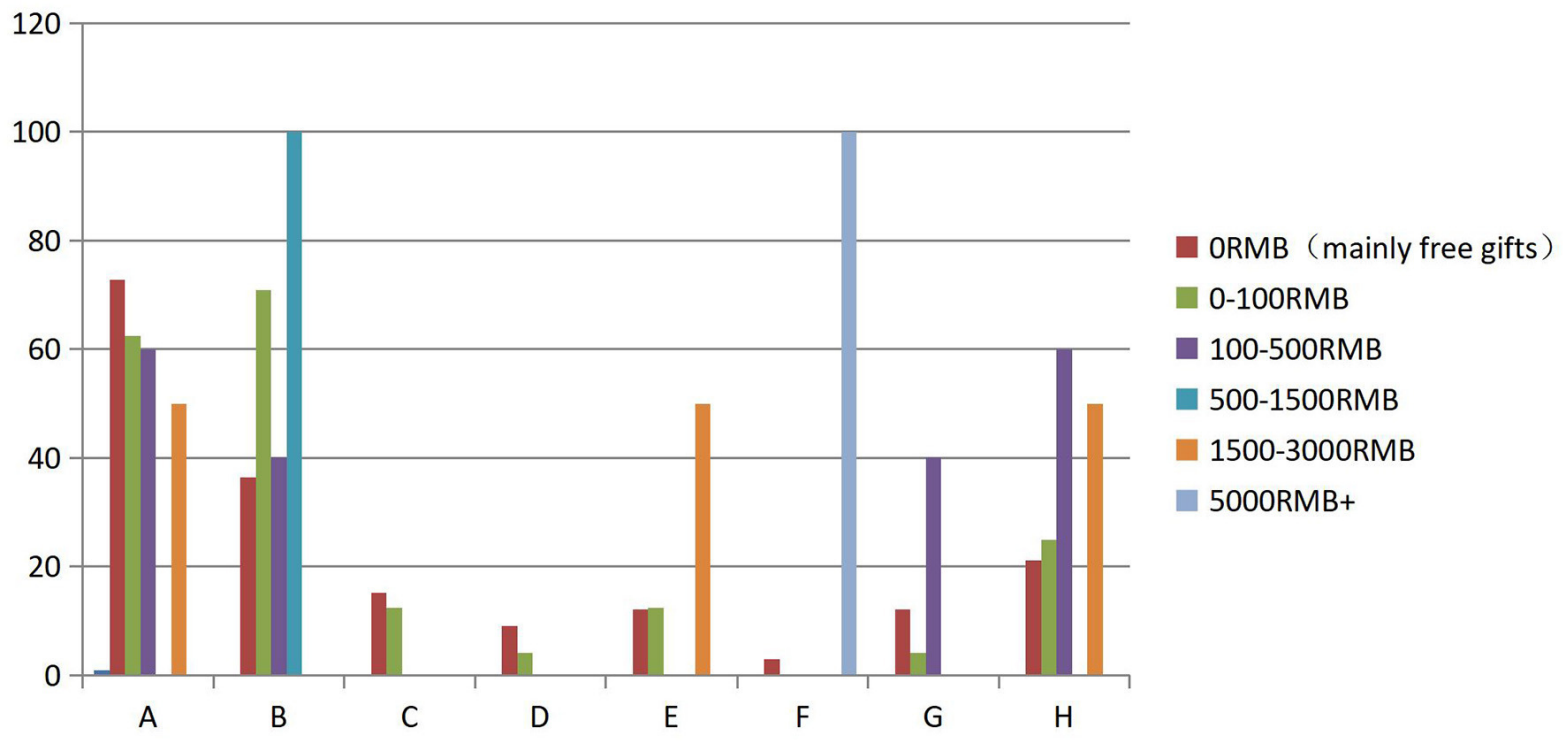
Cross summary table. (A) Someone who is Talented. (B) Someone who is authentic. (C) Someone who is sophisticated. (D) Someone who is distant. (E) Someone I admire. (F) Someone who understand me. (G) Someone who is the person I want to meet in person. (H) Someone who is my friend Blue bar is prevalence rates, red bar is response rates (see Table 2 in the Appendix for specific values).

Previous analysis has confirmed the existence of PSR on Douyu, but this study did not find a solid and significant correlation between PSR and viewers' gift-giving behavior. A total of 66 participants reported that they had gift-giving behavior in the past. Half of them expressed that they only gave free gifts (N = 33), and more than a third of participants said they spent between 0 and 100RMB (N = 24) on gifts. The remaining participant's answers were distributed across the remaining four questionnaire options. Since those numbers were too small to be represent, the study did not analyze that data (see Figure 2).

In this cross-analysis, there was a significant difference in the proportion of options selected (χ^2^ = 60.284 *p* = 0.033 < 0.05). For the first group of participants who only gave free gifts, the option that received the most responses was “Someone who is talented”. As noted hereinbefore, this option could not effectively predict PSR, so the high prevalence rate of this option does not prove a correlation between PSR and viewers' gift-gifting behavior.

For the five options that could predict the presence of PSR, the positive perception of the streamer's “authenticity” received the most responses from the first group of participants, with a prevalence rate of more than one-third. The remaining four options did not receive much response, with the highest prevalence rate being only one in five. Thus, there was only a weak to a moderate positive correlation between PSR and gift-giving behavior among viewers who gave all free gifts.

The second option received the most response from viewers whose gifts accumulated in the 0–100 RMB range was the positive perception of the streamer's authenticity, reaching a 70% prevalence rate. Although this option was strongly associated with viewers' gift-giving behavior, the other four prediction options did not show a significant correlation. Therefore, there is only a moderate positive correlation between PSR and the gift-giving behavior of the viewer whose gifts are accumulated at 0–100 RMB.

**Figure 3:**
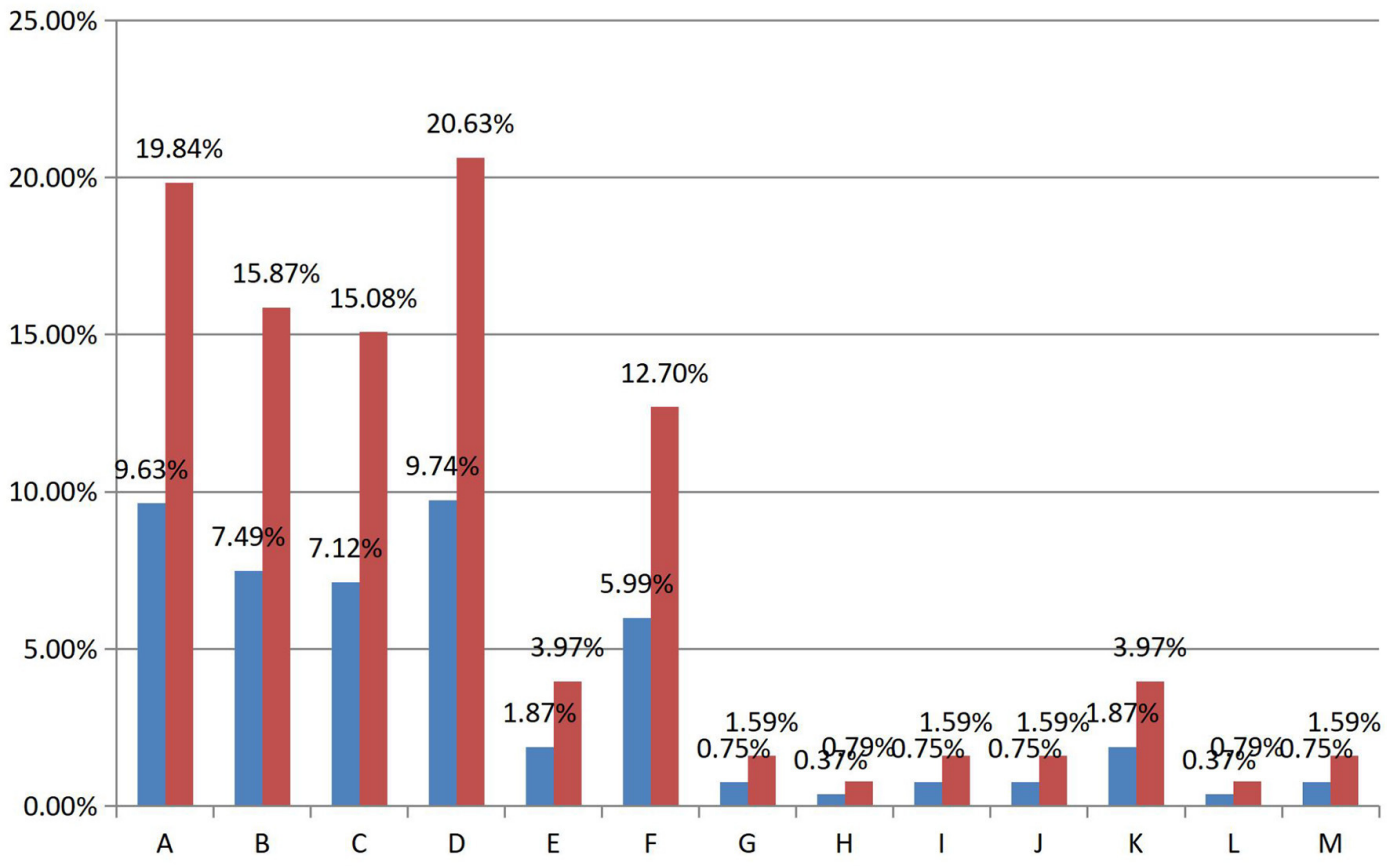
Summary table of response rates and prevalence rates. (A) I want to help the streamer complete the tasks prescribed by Douyu. (B) I want to encourage the streamer to present their live content better. (C) I am impressed by the streamer's skills. (D) I love the streamer's personality. (E) I love the streamer's looks. (F) I love the streamer's style. (G) I feel like the streamer and I are friends. (H) I want the streamer to notice me. (I) I want the streamer to talk to me in the live stream. (J) I see streamers thanking viewers for their gifts; I also want to be publicly thanked. (K) I am in the streamers “fan group,” giving gifts can improve my “fan badge” level. (L) Giving gifts helps me to have a better identity in the streamers' community. (M) I know that expensive gifts can make streamers agree to givers' requests. I want to see the streamer play with my choice of character, weapon, etc (blue bar is prevalence rates, red bar is response rates. See Table 3 in the Appendix for specific values).

Participants' answers to other questions also predicted a correlation between PSR and gift-giving behavior (see Figure 3). There were 13 prediction options in this part, of which six predicted the correlation between social presence and gifting behavior. Among the seven predictors of the correlation between PSR and gift-giving behavior, “I love the streamer's personality” received the most responses from participants, with a response rate of more than one-fifth (N = 126). A similar response rate appeared in the option “I want to help the streamer complete the tasks prescribed by Douyu”. In addition, another two options had an average response rate of around 15 percent: “I want to encourage the streamer to present their live content better”, and “I love the streamer's style”. The total response rate of these four items was half of the total, indicating a significant positive correlation between PSR and gift-giving behavior. However, 201 of the participants indicated that they had not given gifts. Therefore, the questionnaire results reflect a moderate positive correlation between PSR and gift-giving behavior.

Furthermore, the study found that the option “I am impressed by the streamer's skills” was also reflected by participants in this questionnaire section (response rate was 15 percent). Combined with the high response rate of the option “Someone who is talented” mentioned hereinbefore, this study suggests a positive correlation between the viewers gifting behavior and the viewer's appreciation and admiration of game streamer's game technology.

The interview results indicated a weak positive correlation between PSR and gift-giving behavior. Although most interviewees reported their positive perception of PSR in the interview, they did not express the relationship between this perception and their gift-giving behavior. Such as:

“*I love this streamer… I feel like he is like a brother to me… As for I did not send a virtual gift, I personally feel there is no need, and my closeness to the streamer does not drive me to give gifts. I just watch his live streaming more, or pay more attention to his real life.“ (Participant 7)*

Six interviewees (out of 10) presented the same idea. Based on the questionnaire answers of these six interviewees, they all positively felt the existence of PSR. This suggests that, for most viewers, the presence of the PSR only has a weak correlation with their gift-giving behavior. A possible reason may be related to the consumption power of the interviewees because nearly 90% of the participants in this study claimed their monthly income is less than 3000RMB, and they are between 18 and 25 years old. So they may not have a strong consumption power. However, interviewees who gave gifts also said:

”*A big reason for my gift-giving is because I like his live streaming style. I only subscribed him on Douyu, and generally I will only watch his live streaming“ (Participant 2)*

This statement is the same as Yu et al.'s (2018) results, indicating a significant positive correlation between PSR and gift-giving behavior. Nevertheless, in this study, most participants did not send a gift. Therefore, this study considers that PSR does exist on Douyu, but there is only a weak to moderate positive correlation between PSR and viewers' gift-giving behavior. The first research question was thus answered.

### The correlation between SP and gift-giving behavior on Douyu

**Figure 4:**
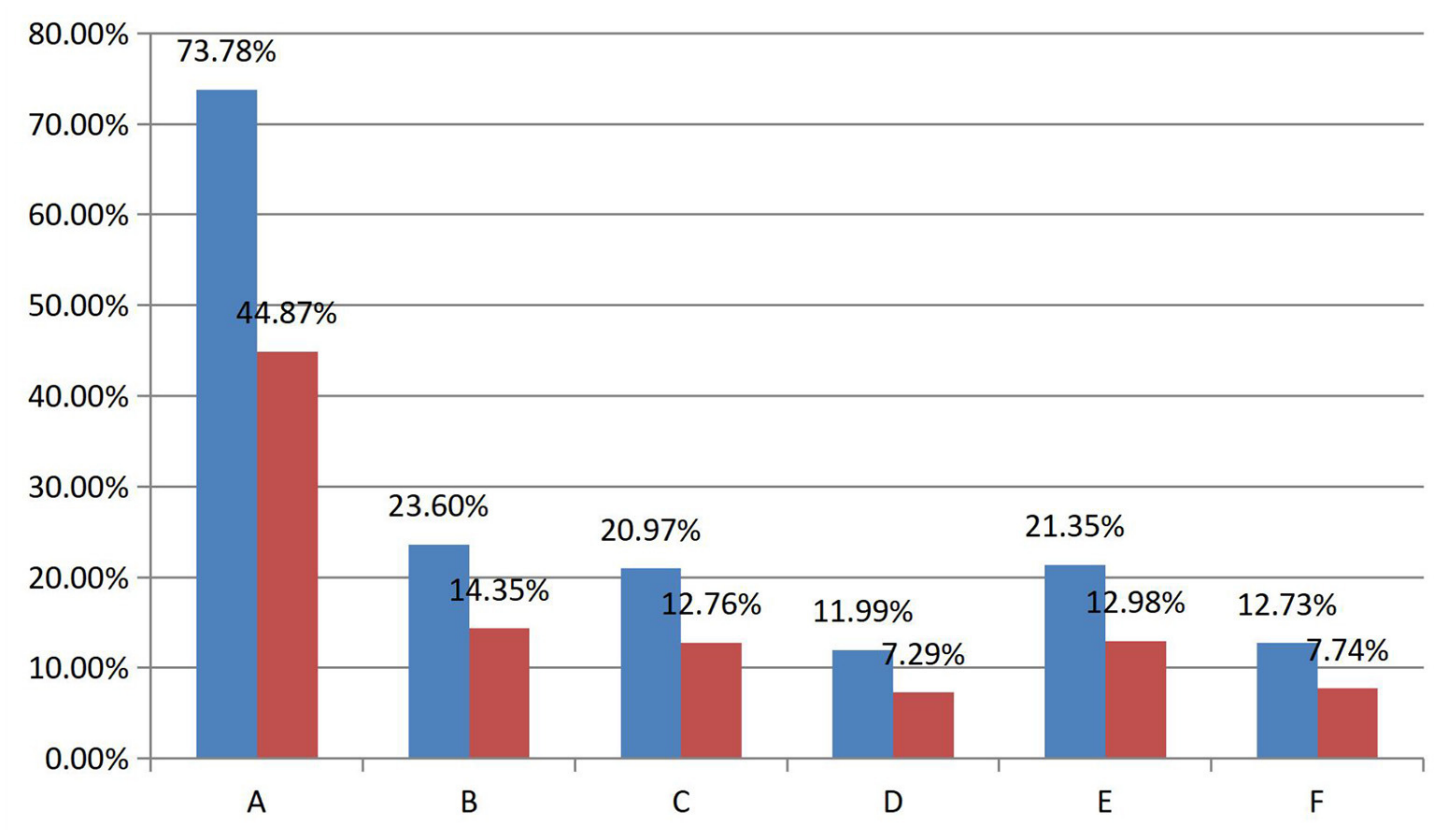
Summary table of response rates and prevalence rates. (A) I spend a lot of time reading other viewers' live comments. (B) I write live comments to connect with other viewers. (C) I write live comments to connect with the streamer. (D) I watch the streamer longer when my comments are replied to (E). I feel that I am actually there in person. (F) I get attention when I see streamers thanking or mentioning virtual gifts or donations from other viewers. (blue bar is prevalence rates, red bar is response rates. See Table 4 in the Appendix for specific values).

This study found that viewers experience different SP when watching live streaming (see Figure 4). In the questionnaire, the SP prediction option that received the highest response from viewers– “I spend a lot of time reading other viewers' live comments”, had a response rate of more than 40 percent (*N* = 439). Although the average response rate of the other five prediction options is not more than 15%, these options are actively predicting the viewers' SP experience, so this study assumes that the viewers can perceive a large number of SP while watching live streaming.

As for the correlation between SP and gift-giving behavior, the researcher found only a very weak association between SP and gift-giving behavior. In this research, six options in the questionnaire predicted the correlation between SP and gift-giving behavior. However, these six options did not have much response rate (see Figure 3). The highest response rate among them was only four percent. Compared with participants' reports on the correlation between PSR and gift-giving behavior, participants' reports on the correlation between SP and gift-giving behavior are infrequent. Consequently, the correlation between SP and gift-giving behavior was weaker than that between PSR and gift-giving behavior, and it was not significant in this research.

The researcher questioned the participants in the following interview. Most of the participants who had gift-giving behavior indicated that SP was not what motivated them to give gifts to game streamers. Instead, their perception of SP motivated them to participate in other live streaming elements. Participants who had not given gifts expressed the same opinion. As one wrote:

”*I see the streamer interacting with other viewers and it actually inspires me to get involved, and I want the streamer to talk to me, but I'm not going to give him a gift just to have him interact with me… And that will make me watch a little bit more… As for wanting to reflect my identity in the follower community, I don't think this is the motivation for me to give gifts “(Participant 10)*

Therefore, this study believes that there is a significant positive correlation between the majority of viewers' live streaming participation and SP, such as sending real-time comments. However, gift-giving is only weakly correlated with SP. Thus, the second research question has been answered.

## Conclusion

In conclusion, this study explored the correlation between viewers' gift-giving behavior and PSI/SP on Douyu by using mixed methods of survey and interviews. Through the analysis of the results, it was found that, first of all, PSR does exist on Douyu, but there is only a weak to a moderate positive correlation between PSR and viewers' gift-giving behavior. Moreover, this study found a certain degree of positive correlation between viewers' appreciation of game streamers' game skills and viewers' gift-giving behavior. Finally, this study also found that although viewers perceived different experiences about SP in the live streaming, there was little to no correlation between SP and audience gift behavior.

Different from previous research, although PSR and SP in this research showed positive impacts on other viewers' live streaming participation, they do not have a significant correlation with gift-giving behavior. One possible reason is that participants in this research were mainly in the age range of 18–25. A large percentage of them were students whose consumption power was limited. Future research could further refine age stratification based on the average graduation ages for different degrees. This research also has other limitations. First, the participants were primarily women, so that the data may lack representation. Second, this research only studied Douyu, a specific platform. Future studies can conduct a mixed study on different Chinese live streaming platforms to interpret the gift-giving behavior of the Chinese viewers in a more macroscopic way.

However, this research innovatively adopted mixed methods to study the relationship between different social relations and gift-giving behavior in live streaming. Although researchers have conducted research in this domain, most of them have not studied China's live streaming platforms. This research still fills the gap in current research on the Chinese viewers' gift-giving behavior and provides a potential direction for future game live streaming studies.

## Challenges and future directions

While the study was completed, the researcher also identified challenges in this research direction. First, Chinese streaming platforms have different styles and elements from those in Europe and the United States, such as Twitch. Future research can explore the gift-giving behavior in game live streaming in combination with the platform economy of Chinese live streaming platforms. Second, as mentioned hereinbefore, the graduation time of students with different degrees may lead to differences in their consumption power. Future studies could break down age groups, or divide participants between 18 and 30 into whether they are working or not.

## Data availability statement

The original contributions presented in the study are included in the article/Supplementary material, further inquiries can be directed to the corresponding author.

## Ethics statement

Ethical review and approval was not required for the study on human participants in accordance with the local legislation and institutional requirements. Written informed consent from the patients/participants or legal guardian/next of kin was not required to participate in this study in accordance with the national legislation and the institutional requirements.

## Author contributions

The conceptualization, data curation, formal analysis, investigation, methodology, project administration, resources, software, supervision, validation, visualization, roles/writing—original draft, and writing—review and editing of this paper were all completed by the WZ.

## Conflict of interest

The author declares that the research was conducted in the absence of any commercial or financial relationships that could be construed as a potential conflict of interest.

## Publisher's note

All claims expressed in this article are solely those of the authors and do not necessarily represent those of their affiliated organizations, or those of the publisher, the editors and the reviewers. Any product that may be evaluated in this article, or claim that may be made by its manufacturer, is not guaranteed or endorsed by the publisher.
