# Supplementary material for: Understanding gift-giving in game live streaming on Douyu: An evaluation of PSR/social presence
Source: Front Psychol. 2022 Aug 11;13:953484. doi: 10.3389/fpsyg.2022.953484 (PMC9403413; doi:10.3389/fpsyg.2022.953484)
Supplement: Supplementary file 1 [file Table_1.docx]

Appendix:

(Table 1) Summary table of response rates and prevalence rates

| Options | response rate | | prevalence rate（n=267） |
| --- | --- | --- | --- |
|  | *n* | response rate |  |
| Someone who is Talented | 171 | 34.69% | 64.04% |
| Someone who is authentic | 108 | 21.91% | 40.45% |
| Someone who is sophisticated | 36 | 7.30% | 13.48% |
| Someone who is distant | 23 | 4.67% | 8.61% |
| Someone I admire | 42 | 8.52% | 15.73% |
| Someone who understand me | 14 | 2.84% | 5.24% |
| Someone who is the person I want to meet in person | 28 | 5.68% | 10.49% |
| Someone who is my friend | 71 | 14.40% | 26.59% |
| Total | 493 | 100% | 184.64% |
| Goodness of fit test：χ²=326.716 *p*=0.000 | | | |

| （Table 2）Cross Summary Table | | | | | | | | |
| --- | --- | --- | --- | --- | --- | --- | --- | --- |
| Options | How much did you spend on virtual gifts for game streamers approximately on Douyu: （%） | | | | | | | Total（*n*=267） |
|  | -3.0（*n*=201） | 0RMB（mainly free gifts）（*n*=33） | 0-100RMB（*n*=24） | 100-500RMB（*n*=5） | 500-1500RMB（*n*=1） | 1500-3000RMB（*n*=2） | 5000RMB+（*n*=1） |  |
| Someone who is Talented | 128（63.68） | 24（72.73） | 15（62.50） | 3（60.00） | 0（0.00） | 1（50.00） | 0（0.00） | 171（64.04） |
| Someone who is authentic | 76（37.81） | 12（36.36） | 17（70.83） | 2（40.00） | 1（100.00） | 0（0.00） | 0（0.00） | 108（40.45） |
| Someone who is sophisticated | 28（13.93） | 5（15.15） | 3（12.50） | 0（0.00） | 0（0.00） | 0（0.00） | 0（0.00） | 36（13.48） |
| Someone who is distant | 19（9.45） | 3（9.09） | 1（4.17） | 0（0.00） | 0（0.00） | 0（0.00） | 0（0.00） | 23（8.61） |
| Someone I admire | 34（16.92） | 4（12.12） | 3（12.50） | 0（0.00） | 0（0.00） | 1（50.00） | 0（0.00） | 42（15.73） |
| Someone who understand me | 12（5.97） | 1（3.03） | 0（0.00） | 0（0.00） | 0（0.00） | 0（0.00） | 1（100.00） | 14（5.24） |
| Someone who is the person I want to meet in person | 21（10.45） | 4（12.12） | 1（4.17） | 2（40.00） | 0（0.00） | 0（0.00） | 0（0.00） | 28（10.49） |
| Someone who is my friend | 54（26.87） | 7（21.21） | 6（25.00） | 3（60.00） | 0（0.00） | 1（50.00） | 0（0.00） | 71（26.59） |
| Goodness of fit test：χ²=60.284 *p*=0.033 | | | | | | | | |

| (Table 3) Summary table of response rates and prevalence rates | | | |
| --- | --- | --- | --- |
| Option | response rate | | prevalence rate（*n*=267） |
|  | *n* | response rate |  |
| I want to help the streamer complete the tasks prescribed by Douyu. | 25 | 19.84% | 9.36% |
| I want to encourage the streamer to present their live content better. | 20 | 15.87% | 7.49% |
| I am impressed by the streamer’s skills | 19 | 15.08% | 7.12% |
| I love the streamer’s personality | 26 | 20.63% | 9.74% |
| I love the streamer’s looks | 5 | 3.97% | 1.87% |
| I love the streamer’s style | 16 | 12.70% | 5.99% |
| I feel like the streamer and I are friends. | 2 | 1.59% | 0.75% |
| I want the streamer to notice me | 1 | 0.79% | 0.37% |
| I want the streamer to talk to me in the live stream. | 2 | 1.59% | 0.75% |
| I see streamers thanking viewers for their gifts; I also want to be publicly thanked. | 2 | 1.59% | 0.75% |
| I am in the streamers “fan group,” giving gifts can improve my “fan badge” level. | 5 | 3.97% | 1.87% |
| Giving gifts helps me to have a better identity in the streamers’ community. | 1 | 0.79% | 0.37% |
| I know that expensive gifts can make streamers agree to givers’ requests. I want to see the streamer play with my choice of character, weapon, etc.. | 2 | 1.59% | 0.75% |
| Total | 126 | 100% | 47.19% |
| Goodness of fit test：χ²=120.175 *p*=0.000 | | | |

| (Table 4) Summary table of response rates and prevalence rates | | | |
| --- | --- | --- | --- |
| Options | response rates | | prevalence rates（*n*=267） |
|  | *n* | response rate |  |
| I spend a lot of time reading other viewers’ live comments. | 197 | 44.87% | 73.78% |
| I write live comments to connect with other viewers | 63 | 14.35% | 23.60% |
| I write live comments to connect with the streamer | 56 | 12.76% | 20.97% |
| I watch the streamer longer when my comments are replied to | 32 | 7.29% | 11.99% |
| I feel that I am actually there in person. | 57 | 12.98% | 21.35% |
| I get attention when I see streamers thanking or mentioning virtual gifts or donations from other viewers. | 34 | 7.74% | 12.73% |
| Total | 439 | 100% | 164.42% |
| Goodness of fit test：χ²=262.727 *p*=0.000 | | | |
